# Anodic Polarity Minimizes Facial Nerve Stimulation as a Side Effect of Cochlear Implantation

**DOI:** 10.1007/s10162-022-00878-8

**Published:** 2022-12-02

**Authors:** Wiebke S. Konerding, Peter Baumhoff, Andrej Kral

**Affiliations:** 1 Department of Experimental Otology, Hannover Medical School, Nife Stadtfelddamm 34, 30559 Hannover, Germany; 2 Cluster of Excellence “Hearing 4 All” (DFG Exc. 2177), Hannover, Germany

**Keywords:** Cochlear implant, Facial nerve stimulation, Electrically evoked compound action potential, Polarity effect

## Abstract

One severe side effect of the use of cochlear implants (CI) is coincidental facial nerve stimulation (FNS). Clinical methods to alleviate FNS range from the reprogramming of processor settings to revision surgery. We systematically assessed different changes in CI stimulation modes that have been discussed in the literature as “rescue factors” from FNS: electrode configuration (broad to focused), pulse shape (symmetric biphasic to pseudo-monophasic), and pulse polarity (cathodic to anodic). An FNS was assessed, based on electrophysiological thresholds, in 204 electrically evoked compound action potential (eCAP) input/output functions recorded from 33 ears of 26 guinea pigs. The stimulation level difference between auditory nerve eCAP threshold and FNS threshold was expressed as the eCAP-to-FNS offset. Coincidental FNS occurred in all animals and in 45% of all recordings. A change from monopolar to focused (bipolar, tripolar) configurations minimized FNS. The Euclidean distance between the CI contacts and the facial nerve explained no more than 33% of the variance in FNS thresholds. For both the FNS threshold and the eCAP-to-FNS offset, the change from cathodic to anodic pulse polarity significantly reduced FNS and permitted a gain of 14–71% of the dynamic range of the eCAP response. This “anodic rescue effect” was stronger for pseudo-monophasic pulses as compared to the symmetric biphasic pulse shape. These results provide possible mechanisms underlying recent clinical interventions to alleviate FNS. The “anodic-rescue effect” may offer a non-invasive therapeutic option for FNS in human CI users that should be tested clinically, preferably in combination with current-focusing methods.

## Introduction

Coincidental facial nerve stimulation (FNS) is an adverse side effect of the therapy of hearing loss using cochlear implants (CI; [1–3]). Some FNS remains unobservable and unnoticed by CI users, whereas clinical signs of FNS range from simple awareness, unpleasant twitches or spasms of the facial muscles, to painful sensations, especially during the use of high current levels [4]. FNS has commonly been reported in about 7% of CI users [4, 5]. The reports range, however, from 1% [2, 6] to 15% [3, 7], with incidences even up to 59% when based on objective, electrophysiological methods [8].

FNS may occur directly after CI activation, but sometimes starts with a time delay of up to several years of CI usage [7, 9]. The FNS is due to the anatomical proximity of the intrascalar CI contacts to the facial nerve (FN), a proximity that allows the current to reach the FN. The distance between the FN canal and the scala tympani (i.e., CI implantation site) is only 240 ± 140 µm [10], and the median distance from the FN to the CI electrode is 1.4 mm, but slightly smaller in CI users that show FNS (1.25 mm; [11]). Nonetheless, most CI users do not report clinical signs of FNS. The major risk factors for FNS are changes that facilitate the current spread beyond the cochlea. As reviewed by Pires et al. [12] these include decreased bone resistance (e.g., in cochlear malformations, otosclerosis, post-meningitis, otosyphilis, temporal bone fracture, and osteoporosis), a low impedance pathway in the modiolar base (e.g., deficient cochleostomy sealing), and the necessity of high CI stimulation levels (e.g., in hypoplastic acoustic nerves or long-term hearing deprivation). The patients’ ages and certain etiologies (e.g., meningitis, encephalitis, early onset SNHL) have been found to be associated with higher incidences of FNS; however, their mechanisms remain unknown [11] and are likely to be secondary to the higher stimulation levels required by higher hearing thresholds.

Non-invasive clinical approaches to eliminate FNS comprise deactivation of electrode contacts and reprogramming of speech processor settings. These non-invasive approaches usually come with negative consequences for speech understanding. Deactivation of the corresponding electrodes reduces the number of effective channels and may adversely affect auditory outcomes [13]. Increasing the pulse duration to decrease the threshold current levels and current spread may limit the maximal available pulse rate [14]. In cases when several electrode contacts cause FNS, revision surgery may be indicated [15]. Recently, the use of triphasic stimulation has been reported to reduce FNS [16, 17]. The standard of contemporary CI stimulation is the monopolar electrode configuration with cathodic-first pulse polarity and a symmetric biphasic pulse shape [18, 19]. When changing from these cathodic-first, symmetric biphasic pulses to triphasic pulses, the polarity of the assumed spike-eliciting phase was also changed [16, 17]. Although it is known that both phases in a biphasic pulse can elicit a response, for symmetric biphasic pulses, the first phase is usually considered the main spike-eliciting phase, and for triphasic pulses, it is the second phase (i.e., usually anodic; [20]). The comparison of FNS elicited by alternating polarities of a triphasic pulses was carried out in one subject and revealed that the “rescue effect” was greatly reduced for cathodic-second, as compared to anodic-second, triphasic pulses [21]. This indicates that the “rescue effect” of triphasic pulses on FNS is not (only) based on the change in spatiotemporal current spread, but also on the polarity of the spike-eliciting phase. In three recent case studies, describing successful re-implantations to alleviate FNS in three, two, and one CI users [18, 22, 23], several aspects of CI stimulation were discussed as potential “rescue factors” from FNS. These factors were all associated with the change in manufacturer and may act either singly or in combination: electrode configuration (a change from broad/monopolar to focused), pulse shape (symmetric biphasic to pseudo-monophasic), or pulse polarity (cathodic to anodic).

As the mechanisms of FNS are still unresolved, particularly in cases in which the preoperative history is unremarkable and cochlear anatomy is normal [24], we used an animal model to systematically analyze FNS to disentangle the potential impact of different stimulation parameters on FNS. We assessed FNS thresholds in electrically evoked compound action potential (eCAP) recordings in response to different stimulation modes, with different combinations of electrode configuration, pulse shape, and pulse polarity. We compared broad-monopolar and focused (bipolar/tripolar) configurations, assessed the influence of the second phase by comparing symmetric biphasic to pseudo-monophasic (psm) pulse shapes, and analyzed the polarity effect (PE). To account for the fact that the auditory nerve and the facial nerve may be differently susceptible to any of these factors, we analyzed not only the FNS threshold, but also the offset between the eCAP threshold (i.e., auditory nerve) and the FNS threshold for the same recordings.

## Material and Methods

### Animals

To characterize potential influences on FNS, we analyzed 204 eCAP input/output (I/O) functions from 33 ears of 26 guinea pigs (11 male, 15 female). The recordings were performed as control measurements in two studies that were initiated to assess the influence of mechanical microlesions in the inner ear. In study 1 [25], the control measurements were performed prior to lesioning (*n* = 25 ears). In study 2, the animals were lesioned in one ear, and the data, reported here, were measured one week later on the non-lesioned, control ears (*n* = 8 ears).

All procedures were in accordance with the German and European Union guidelines for animal welfare (ETS 123, EU Directive 2010/63/EU) and were approved by the German state authority (Lower Saxony state office for consumer protection and food safety, LAVES approval No. 14/1514 and 18/2789).

### Surgical Preparation

Anesthesia was induced by subcutaneous ketamine/xylazine injections (50 mg/kg ketamine, 10 mg/kg xylazine, with 0.1 mg/kg atropine sulfate). For subsequent inhalation anesthesia, a custom-made endotracheal tube was inserted through a tracheotomy and connected to a ventilator (Rodent Ventilator 7025, Ugo Basile, Comerio, Italy). After surgical preparation, an adequate anesthesia level was maintained by < 1.5% isoflurane in a mixture of O_2_/air and was monitored by testing for paw-withdrawal reflexes. Additional local anesthesia (2% lidocaine) was applied throughout the surgery as needed. Vital functions were assessed by electrocardiography and capnometry (end-tidal CO_2_ vol%; Normocap CO_2_ & O_2_ Monitor, Datex, Helsinki, Finland). Body core temperature was kept at around 38.0 °C, using a heating pad controlled via feedback from a rectal temperature probe (TC-1000 Temperature Controller, CWE Inc., Ardmore, USA). To prevent dehydration, Ringer’s solution was supplied via a continuous subcutaneous infusion (2 ml/h).

Anesthetized animals were fixed in a stereotaxic frame (Stereotaxic Frame 1430, David Kopf Instruments, Tujunga, USA; custom, stainless steel fixation rod). The pinna and the overlying soft tissue were removed, and the bulla was carefully opened, providing access to the basal cochlear turn and round window. A cochleostomy was drilled into the scala tympani (0.6 mm diameter, 4000 rpm) at the basal cochlear turn, approximately 1 mm ventral to the round window. For eCAP recordings, a small silver ball electrode (ø ~ 500 μm) with Teflon-insulated shaft was placed in close contact with the round window membrane. Care was taken that all middle ear structures remained undamaged. As a reference, a subcutaneous Ag/AgCl electrode was placed retroauricularly. Normal hearing was confirmed by auditory brainstem responses using alternating condensation and rarefaction clicks (50 μs) averaged over 100 repetitions. To prevent any confounding influences due to electrical stimulation of hair cells, the animals were acutely deafened via a neomycin sulfate infusion (Caesar & Lorentz GmbH, Hilden, Germany, 10% in saline) into scala tympani (for details see [25]).

### Cochlear Implant Stimulation

**Fig. 1:**
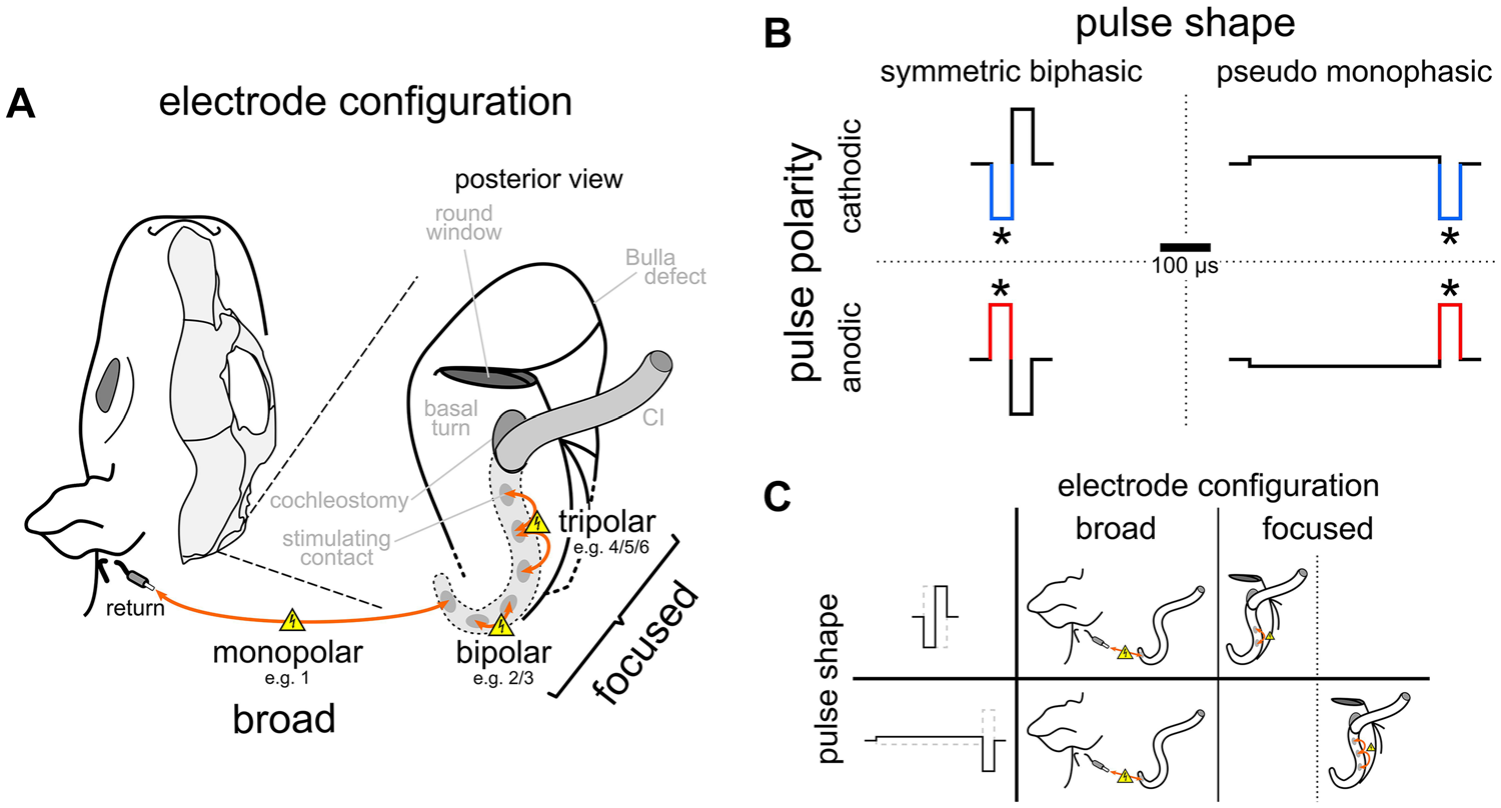
Schematic drawing of the different cochlear implant (CI) stimulation modes applied during the study. The stimulation mode was varied both with respect to the electrode configuration (**A**) and the pulse shape (**B**), with alternating presentation of the pulse polarity of the assumed spike-eliciting phase (asterisks). **C** Stimulation modes were 4 different combinations of electrode configuration and pulse shape: (1) We combined the monopolar configuration with both the symmetric biphasic and the pseudo-monophasic (psm) pulse shape, and (2) the two focused configurations were paired with both pulse shapes, which are bipolar-symmetric biphasic and tripolar-psm. The stimulation for each stimulation mode was performed at different, but corresponding CI contacts, i.e., from most basal to most apical stimulation contacts

Guinea pig-adjusted CIs (6 contacts; electrode spacing, 700 μm; diameter, 0.5 mm; contact impedance, ~ 21 kΩ; full insertion, 5 mm; MedEl comp., Innsbruck, Austria) were implanted through the cochleostomy and used for stimulation. All stimulations and recordings were performed using a custom-built electrophysiology setup (Otoconsult Comp., Fankfurt/M., Germany) and remotely controlled by a computer with a 96-channel DIO card (PCIe 6509, National Instruments, Austin, USA). The stimuli were computer-generated and transmitted by a 32-bit MIO card (PCIe 6259, National Instruments; output sampling rate: 2.8 MHz). The stimulation current was generated by optically coupled constant current sources (ICS 10, Otoconsult Comp., Frankfurt/M., Germany) and attenuated from the maximal output of 10 mA in decibel (dB) steps (ATT 15 attenuator, Otoconsult Comp.). All electrical stimuli were charge-balanced and were delivered in one of three different electrode configurations: monopolar, bipolar, or tripolar (Fig. 1A), with symmetric biphasic or psm pulse shapes (Fig. 1B). Stimulation was performed using 50-µs-long phases (no inter-phase gap) of alternating polarity (150 or 200 repetitions, each), starting with the anodic spike-eliciting phase, that is, anodic-first in symmetric, biphasic pulses and cathodic-first in asymmetric, psm pulses (Fig. 1B). The psm pulses were prepared in an 8:1 ratio, with an 8-times longer initial phase. The 50-µs-long, second phase with an 8-times larger current amplitude was expected to be the spike-eliciting phase (see “Discussion”). For the broad stimulation, a monopolar configuration was achieved via a subcutaneous return electrode (contralateral ear; for details, see [25]). For the focused stimulation, we used two common electrode configurations: bipolar and tripolar stimulation. Due to time constraints, we could not test each combination of focused configuration with pulse shape, but paired symmetric biphasic pulses with bipolar configuration and psm pulses with tripolar configuration (Fig. 1C). In the bipolar configuration, two CI contacts were used as stimulation and return electrodes. Alternating polarity changed the direction of current flow in the apical-basal direction. The leading phase in bipolar stimulation was defined as the phase of the more apical contact. For focused stimulation in the tripolar configuration, three neighboring CI contacts were used, with the middle CI contact used as the stimulating electrode. This was achieved by combining two current sources that used the same working contact at the center electrode and two flanking return electrodes with equal return current (0.5 σ, [26]). The rate of presentation was ~ 30 Hz (33 ms recording interval).

Each stimulation mode and CI contact combination was repeated at several current levels to determine the eCAP threshold and the FNS threshold. For this, the current was elevated in 1-dB steps from a subthreshold level to an average of 10 dB above the visually detected eCAP threshold, usually resulting in 21 eCAP steps per I/O function. As we chose the stimulation range relative to the eCAP thresholds (online, during alternating stimulation), the maximal current levels differed significantly, based on stimulation mode (Kruskal–Wallis test: *H*(3,204) = 99.54, *p* < 0.0001, *η*^*2*^ = 0.4827) ranging from, on average, 959 µA (monopolar-psm) to 4131 µA (tripolar-psm). However, the range from eCAP threshold to the maximal current level applied was comparable with on average 10 dB above the eCAP threshold, with no significant difference based on polarity. The significant difference between stimulation modes (2-way ANOVA: polarity (*F*(1,307) = 2.134, *p* = 0.1451, *η*^*2*^ = 0.0069); mode (*F*(3,307) = 7.197, *p* < 0.0001, *η*^*2*^ = 0.0657); interaction (*F*(3,307) = 1.092, *p* = 0.3525, *η*^*2*^ = 0.0106)) was based on the significantly lower current range in the cathodic monopolar-psm mode (7 ± 4 dB) and a significantly higher current range in the anodic tripolar-psm mode (13 ± 8 dB) than in the other stimulation modes of a given polarity (monopolar-symmetric biphasic anodic and cathodic, 10 ± 2 dB; bipolar-symmetric biphasic anodic and cathodic, 8 ± 4 dB; monopolar-psm anodic, 9 ± 4 dB; tripolar-psm cathodic, 11 ± 6 dB). These differences were, however, in the opposite direction to those observed in the data: They would reduce the likelihood of detecting FNS in the cathodic monopolar-psm mode (which had the highest numbers of detected FNS; see Fig. 4) and would elevate the likelihood of detecting FNS in the anodic tripolar-psm mode (which had the lowest numbers of detected FNS; see Fig. 4). Thus, the range of maximal current levels applied had no confounding effect on our results.

### Electrically Evoked Compound Action Potential Recordings

Recordings from left and/or right ears were carried out in anesthetized animals in a sound-attenuating chamber (for details see [25]). The eCAP responses were recorded from a silver ball electrode in contact with the round window membrane that was referenced to a subcutaneous Ag/AgCl electrode, placed retroauricularly at the ipsilateral ear (recording impedance <30kΩ). The recording was performed using custom software (AudiologyLab, Otoconsult Comp.) through a 32-channel MIO card (NI-6259, National Instruments) with a sampling frequency of 250 kHz.

### Analysis

Automatic Matlab procedures were used to analyze the eCAP recordings for assessing both the eCAP and FNS thresholds. The single sweeps were averaged offline, after separating the two polarities (*n* = 150–200 sweeps). In total, *n* = 408 FNS threshold-measurements were conducted based on the eCAP I/O functions: symmetric biphasic-monopolar (*n* = 198), psm-monopolar (*n* = 88), symmetric biphasic-bipolar (*n* = 62), and psm-tripolar (*n* = 60). Stimulation was performed using different CI contacts, with equal proportions of the most apical (#1 or #2) and the most basal contacts (#5 or #6) in all stimulation modes (see “Discussion”).

**Fig. 2:**
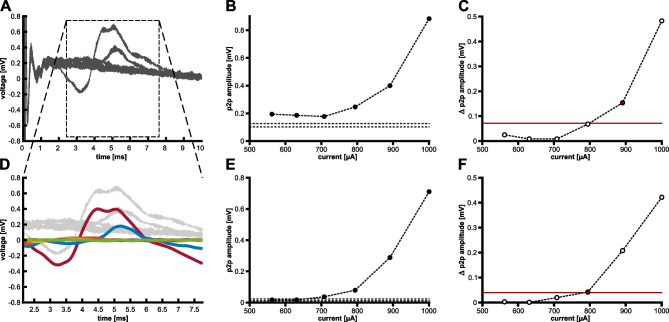
Introduction of pre-processing steps refined the automatic facial nerve stimulation (FNS) detection. **A–C** The top row shows FNS detection based on unprocessed data. **D–F** The bottom row shows the FNS detection after pre-processing (i.e., filtering and detrending). **A, D** As a representative example, we show averaged responses for different current levels (colored lines in **D**) to a monopolar, anodic-first biphasic stimulation at an apical CI contact #1 (male guinea pig). **B, E** The maximal peak-to-peak (p2p) amplitude of the traces given in panels (**A**) and (**D**), respectively, is always above the baseline level (min–max: dashed lines) for unprocessed (**B**), but not for pre-processed data (**E**). **C**, **F** The difference between p2p values (Δ p2p) of subsequent stimulation levels, shown in panels (**B**) and (**E**), crosses the threshold criterion for FNS threshold detection (horizontal, red line) at different current levels, resulting in a lower FNS threshold-value (red symbol) for pre-processed (**F**) than for unprocessed data (**C**)

The FNS threshold analysis used a time window (2.1–7.7 ms) previously described for FNS responses in eABR [8] and eCAP recordings [27, 28]. We furthermore confirmed via visual inspection of the online averaged signal and a live video stream of the animal that the occurrence of a response in this time window coincided with twitching and whisker movements ipsilateral to the side of CI stimulation. The time window was adjusted to the onset of the assumed spike-eliciting phase (Fig. 1). The recordings were pre-processed via smoothing (Savitzky-Golay filter, 4th order, frame length: 179 samples) and detrending. Such pre-processing guaranteed reliable FNSthreshold detection (Fig. 2A–C for threshold detection from unprocessed data). We subsequently determined the peak-to-peak (p2p) amplitude of the FN response for each stimulation current (colored lines; Fig. 2D), using minimum and maximum values within the given latency range (Fig. 2E). The threshold-detection criterion for FNS was based on the assumption that the change in p2p amplitude with rising current would increase and exceed the value of background fluctuations in p2p amplitude when the FNS threshold was reached. Thus, we calculated the differences between p2p amplitudes of successive current levels (delta p2p) and used the delta values of the 6 lowest current levels as a baseline (these were always below the eCAP and FNS threshold). The FNS threshold criterion (red line; Fig. 2F) was defined as delta p2p larger than the mean plus 8 times the standard deviation (SD) of the baseline (Fig. 2F).

We also compared the FNS threshold to the eCAP threshold (i.e., auditory nerve) using the same eCAP recordings, but within a different time window: between 0.62 and 1.62 ms after stimulus onset. As described previously [25], this is the time window for the late eCAP components (N2P2) that is free of electrical artifacts. The data were averaged for each polarity, and pre-processed using Matlab procedures: 3rd order sgolay filter with a 63-sample frame length and detrending. We used sigmoidal fitting to assess the parameters of the I/O function and included only measurements with high goodness of fit (*r*^2^ ≥ 0.8). The threshold was defined as the current level that led to 10% of the maximal (i.e., fitted) p2p amplitude. Based on these eCAP thresholds, we calculated the individual eCAP-to-FNS offsets as the difference between eCAP and FNS threshold (in dB) of the same recording (1). Positive values thus indicate that the FNS threshold was higher than the eCAP threshold (i.e., the expected outcome).

1 $$FNS \; offset=FNS \;threshold \left[dB\right]-eCAP \;threshold \left[dB\right]$$

To assess the difference between anodic and cathodic stimulation, we calculated the pairwise difference between the respective threshold values as the polarity effect (PE, Formula 2). This was carried out for both the FNS threshold (threshold PE) and the eCAP-to-FNS offset (offset PE) in dB (2). Positive values thus indicate higher anodic than cathodic values.

2 $$PE=anodic [dB\; attenuation]-cathodic [dB\; attenuation]$$

To calculate a threshold PE for those cases where only one polarity induced a detectable FNS within the current-level range tested, we approximated the FNS threshold of the other polarity as 1 dB above the highest stimulation current tested (approximated thresholds). This approach underestimated the effect size of the PE (see “Discussion” section).

### Anatomical Evaluation

Post mortem, all cochleae were fixed in 3.5–3.7% methanal solution. In a subset of 17 temporal bones (13 left and 4 right), the CIs were left in situ. Subsequently, high resolution 3-dimensional (3D) DICOM data sets of all specimens were obtained by performing quantitative micro-computed tomography (µCT, Xtreme CT II, SCANCO Medical AG, Brüttisellen, Switzerland). The µCT used settings of 68 kVp, 1470 µA, 100 W, and at voxel size of 17 µm. After the first scan, the CIs were removed from the specimens, and the scans were repeated using the same settings. This additional scan helped avoid obscuring bone structures by blooming artifacts caused by the metal CI contacts. All DICOM images were processed using the visualization platform AMIRA™ (v6.5, FEI Visualization Sciences Group, Bordeaux, France).

**Fig. 3:**
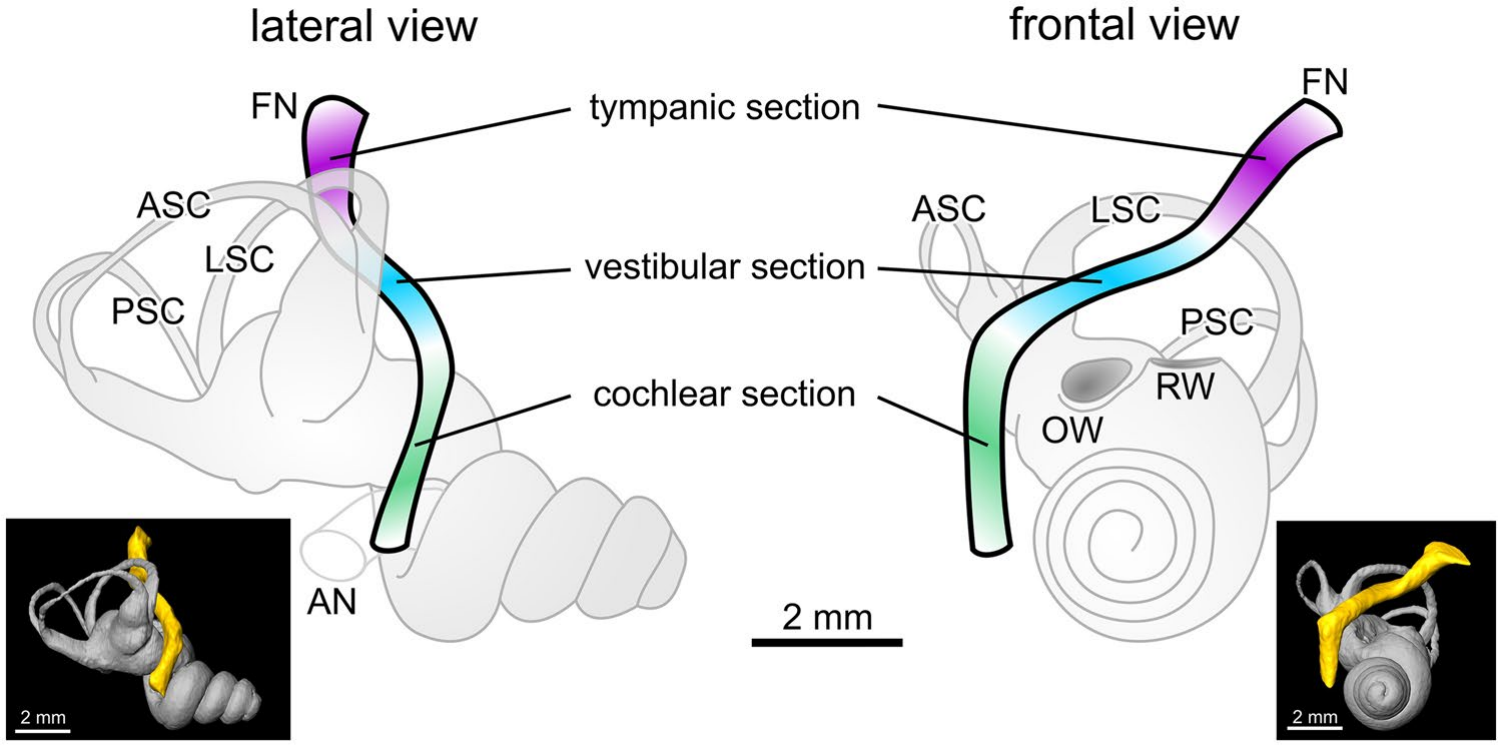
Schematic drawing of the cochlea and facial nerve (FN) with three anatomical sections: cochlear, vestibular, and tympanic. The insets show an example of a segmented FN relative to the template cochlea

For a combined analysis of all cochleae, a template cochlea was generated from 10 scans (5 animals) without CI: right cochleae (*n* = 5) were mirrored. We found only little variation in cochlear size and anatomy, thus enabling an average reconstruction of Rosenthal’s canal, the fluid spaces, and the modiolar tract of the auditory nerve. The temporal bones of all 17 specimens of the present study were then registered to the template cochlea, again mirroring the right specimens (see Fig. 9). We marked the center positions of all CI contacts using the filamenting module of the visualization platform (bright on dark, linear connections) and transferred them onto the template. The FN tract was reconstructed from the µCT images, taken after removing the CI. The midpoints of the facial nerve canal were marked (mean: 31 points; 21–43 points, depending on reconstruction) in the filamenting module (dark on bright), starting at the disjunction of the FN from the vestibulocochlear nerve at the internal meatus. The starting points of the FN reconstructions were visually determined from sagittal slices. They showed little inter-individual variation from the average coordinates (mean, 170 µm; 30–450 µm). From the 3D coordinates of all FN reconstructions, an average topology was calculated in 0.2 mm segments. These were used to define the 3 sections of the FN (Fig. 3): Cochlear section (0–1.6 mm facial nerve length), vestibular section (2–5.2 mm), and tympanic section (5.6–8.2 mm). We excluded 0.4 mm at the bends between both the cochlear and vestibular sections and the vestibular and tympanic sections. We assessed the minimal distance of individual CI contacts to the FN (CI-to-FN distance) either as an overall minimum or as a separate value for each of the 3 defined FN sections.

### Statistics

Statistical analyses were performed using GraphPad Prism 5 (GraphPad Sofware Inc., San Diego, CA), and all values are given as the mean ± standard deviation. Normality was tested using the Kruskal–Wallis normality test, and non-parametric tests were used where applicable. To assess the influences of two factors (stimulus polarity or CI stimulation contact and stimulation mode), 2-way ANOVAs were performed. We combined pulse shape and configuration as one factor and subsequently disentangled the two aspects of the stimulation mode using Bonferroni corrected post-tests: (A) For pulse shape effect assessment, we compared symmetric biphasic pulses and psm pulses, both with monopolar configuration, and B for assessment of the effect of configuration, we compared monopolar and bipolar stimulation, both using symmetric biphasic pulse shapes. When assessing the effect of stimulation mode, i.e., only on the PE, 1-way ANOVA and the respective post-tests (planned contrast, see above) were used. Where two distributions were compared, we used a paired *t*-test or a Wilcoxon signed rank-sum test (from now “Wilcoxon test”) or, in the case of independent data, the unpaired *t*-test or (Wilcoxon-)Mann–Whitney *U* test. Correlations were assessed using Pearson or Spearman correlation. The significance level was always set to 5%.

## Results

After introducing the pre-processing steps, the automatic FNS detection procedure defined threshold criteria for FNS in all but 2 cases. Cases for which the FNS threshold was detected to be equal or lower than the (auditory nerve) eCAP threshold (*n* = 13) were excluded. The analyses were based on 393 anodic and cathodic eCAP I/O functions. An FNS was detected in 178 of these cases.

### Potential Confounding Factors: Sex and Weight

Based on the assumption that anatomical differences (e.g., bone density or muscle volume) might have had an impact on the susceptibility of the FN to CI stimulation, we assessed the potential influences of sex and weight (i.e., an approximation of age in guinea pigs). Although the males were significantly heavier than the females (Mann–Whitney *U* test: *U*(11,15) = 40.50, *p* = 0.0305), there was no significant sex difference in the minimal FNS threshold off all stimulation modes and polarities (*U*(11,15) = 64.50, *p* = 0.3624), and the body weight did not correlate significantly with the minimal FNS threshold (Spearman correlation: *r* =  − 0.3531, *p* = 0.0768). Thus, the FNS data were merged for subsequent analyses.

### FNS Frequency and Threshold Depends on Electrode Configuration and Polarity

**Fig. 4:**
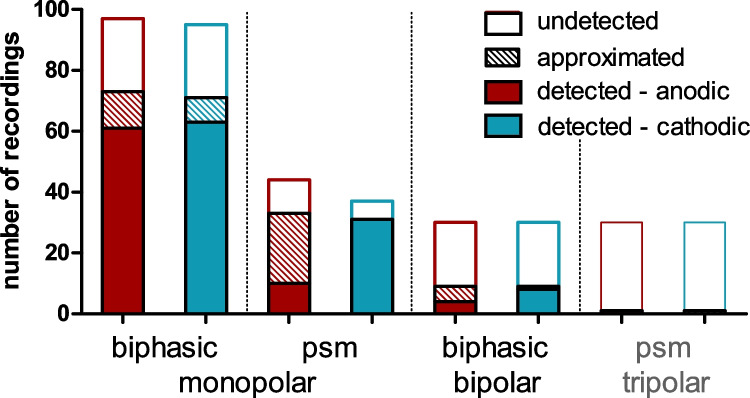
In the majority of recordings (178 out of 393; pooled stimulation contacts), we stimulated up to levels that generated facial nerve stimulation (FNS) responses, at least for one stimulus polarity. Since detected thresholds (filled areas) were more frequent when using cathodic (aqua) rather than anodic (red) stimulation, the results indicate an asymmetry in FNS thresholds, with lower thresholds with cathodic stimulation. Focused configurations (bipolar, tripolar) reduced the occurrence of FNS relative to broad-monopolar stimulation, despite the higher current levels applied. We also indicate the number of approximated FNS (hatched areas) that were subsequently included to calculate the polarity effect of the FNS. Since we only detected one case of FNS in the tripolar configuration (grey lettering), this condition was excluded from further analyses

Broad-monopolar stimulation configurations generated FNS responses more often (biphasic and psm pulses: 65% and 51%) than did focused stimulation (bipolar and tripolar configuration: 20% and 2%). Since we only detected one case of FNS in the tripolar configuration (Fig. 4), this condition was excluded from further analyses.

We detected FNS more frequently in cathodic than anodic stimulation; this was particularly apparent in the monopolar configuration with psm pulses (Fig. 4). Sometimes, the FNS threshold was not reached for one polarity (usually the anodic). To be able to calculate the paired PE (see Figs. 6 and 7C) under these conditions, we approximated the FNS of the other polarity as 1 dB above the stimulation level (Fig. 4; hatched segments). These approximated data were not included in analyses of FNS thresholds beyond paired threshold or offset PEs.

**Fig. 5:**
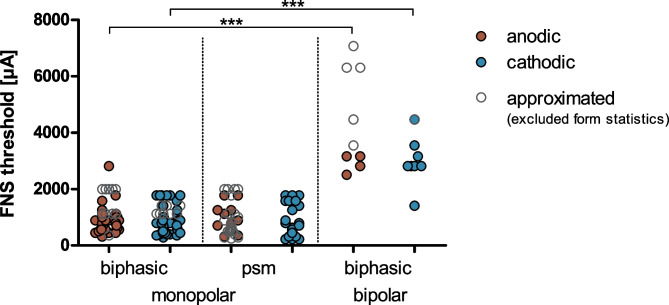
Facial nerve stimulation (FNS) demonstrated significantly higher FNS thresholds for the bipolar than the monopolar configuration (i.e., symmetric biphasic pulses) and significantly higher FNS thresholds for anodic (red) than cathodic polarity (aqua). The stimulation contacts were pooled for the analysis. The approximated values (open circles) were used to calculate the PE, but were not included in the analysis of the FNS thresholds. Two-way ANOVA with post hoc tests: ***p < .001

Averaging both polarities, the FNS thresholds were 4.04 ± 1.71 mA for symmetric biphasic pulses in the bipolar configuration, 1.03 ± 0.46 mA for symmetric biphasic pulses in the monopolar configuration, and 0.96 ± 0.46 mA for psm pulses in the monopolar configuration. In accordance with the differences in FNS prevalence, the detected FNS thresholds differed both in polarity and stimulation mode (2-way ANOVA: polarity (*F*(1,220) = 21.5, *p* < 0.0001, *η*^*2*^ = 0.0891); mode (*F*(2,220) = 308.8, *p* < 0.0001, *η*^*2*^ = 0.7374); interaction (*F*(2,220) = 11.0, *p* < 0.0001, *η*^*2*^ = 0.0907); Fig. 5). The post hoc test revealed significantly higher FNS thresholds for focused than for monopolar stimulation with symmetric biphasic pulses for both polarities (*p* < 0.001) and no difference in FNS threshold between pulse shapes in the monopolar configuration (symmetric biphasic vs. psm: *p* > 0.05; Fig. 5).

**Fig. 6:**
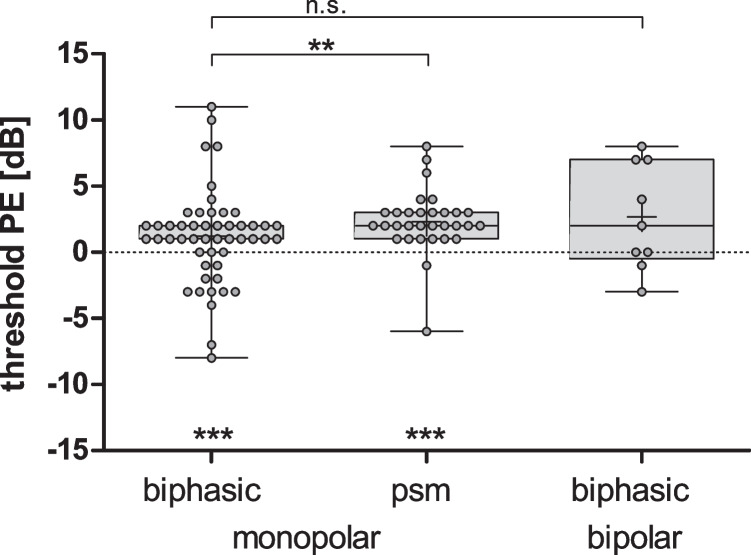
The FNS threshold polarity effect (threshold PE) revealed that anodic stimulation leads to significantly higher FNS thresholds than cathodic stimulation (i.e., positive values). The effect was largest in the pseudo-monophasic (psm)-stimulation configuration. In bipolar-biphasic stimulation, the average threshold PE was positive; this did not, however, reach statistical significance (*n* = 9). Given the approximation necessary for calculation of the PE, the actual PE in all stimulation modes may have had higher absolute values (underestimate of the effect). The stimulation contacts were pooled for the analysis. Shown are boxplots with min–max range (whisker) and means (cross). Wilcoxon test vs. 0: ***p < .001. Kruskal–Wallis test with post-tests: **p < .01

To assess the effect size of the PE, the threshold PE was calculated as a paired difference between anodic and cathodic FNS thresholds. Positive threshold PEs represented higher anodic than cathodic FNS thresholds (Fig. 6). In both monopolar configurations, the threshold PE was significantly positive (Wilcoxon test against zero: symmetric biphasic (*W*(69) = 1168, *p* = 0.0002); psm (*W*(30) = 431, *p* < 0.0001)). There was also a significant effect of pulse shape, with significantly higher threshold PE for psm pulses (2.3 ± 2.4 dB) than for symmetric biphasic pulses (1.2 ± 3.0 dB) in the monopolar configuration (Kruskal–Wallis test with Dunn’s post-test (*H*(2,110) = 8.422, *p* = 0.0148, *η*^*2*^ = 0.0600); symmetric biphasic vs. psm (*p* < 0.01)). Also in the bipolar configuration (symmetric biphasic pulses), the average threshold PE was positive (2.7 ± 4.0 dB). This, however, did not reach statistical significance (*t*-test vs. 0: *T*(8) = 2.000, *p* = 0.805) and was not significantly different from the monopolar configuration (post-test: *p* > .05).

### ECAP-to-FNS Offset Depends on Pulse Shape and Polarity

**Fig. 7:**
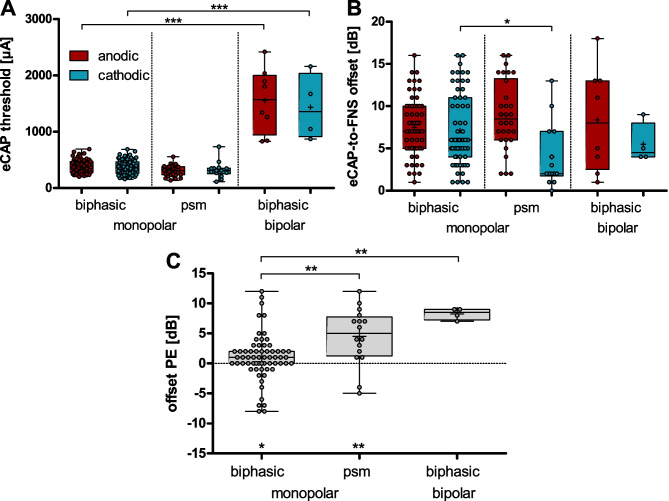
The range of current levels from electrically evoked compound action potential (eCAP) thresholds and facial nerve stimulation (FNS) thresholds were defined as eCAP-to-FNS offset. **A** Whereas the eCAP thresholds were significantly higher in bipolar than in monopolar configuration (i.e., with symmetric biphasic pulses), **B** the eCAP-to-FNS offsets differed for the cathodic polarity, only, with higher offsets for symmetric biphasic than psm pulses (i.e., in monopolar configuration). **C** The offset PE demonstrated that, in all configurations, the anodic polarity significantly increased the available stimulation range (till FNS) relative to the cathodic polarity (i.e., positive values). The stimulation contacts were pooled for the analysis. Two-way ANOVA with post-tests: ***p < .001 and *p < .05. Kruskal–Wallis test with post-tests: **p < .01

The difference between auditory-nerve threshold and FNS thresholds defines the maximal range of current levels available for auditory stimulation with a CI (Fig. 7B). For this reason, we also compared the eCAP thresholds (i.e., auditory nerve) to the FNS thresholds. The eCAP thresholds (Fig. 7A) were distinct for different stimulation modes, but not for polarity (2-way ANOVA: polarity (*F*(1,183) = 0.8609, *p* = 0.3448, *η*^*2*^ = 0.0049); mode *(F*(2,183) = 320.8, *p* < 0.0001, *η*^*2*^ = 0.7780); interaction (*F*(2,183) = 0.86, *p* = 0.4245, *η*^*2*^ = 0.0093)). Post hoc tests revealed a significant effect based on electrode configuration with lower thresholds for symmetric biphasic stimulation in the monopolar (382 ± 123 µA, *n* = 133) than in the bipolar configuration (bipolar-biphasic stimulation: 1560 ± 703 µA, *n* = 12; post-tests: *p* < 0.001). There was no significant difference based on pulse shape in the monopolar-configuration (psm: 323 ± 132 µA, n = 44; post-test: *p* > 0.05).

However, when analyzing the eCAP-to-FNS offset, there was a significant effect based on polarity, but not based on the stimulation mode for the available dynamic range (2-way ANOVA: polarity (*F*(1,183) = 8.596, *p* = 0.0038, *η*^*2*^ = 0.0449); mode (*F*(2,183) = 0.2650, *p* = 0.7675, *η*^*2*^ = 0.0029); interaction (*F*(2,183) = 1.970, *p* = 0.1423, *η*^*2*^ = 0.0211)). On average, the eCAP-to-FNS offsets were 7.3 dB (Fig. 7B) for all 3 stimulation modes. Besides the lack of an effect of stimulation mode for the population analysis, the post hoc analyses revealed a significant effect of the pulse shape, but only for cathodic stimulation: In the monopolar configuration, the eCAP-to-FNS offset was smaller for the psm pulses than for the symmetric biphasic pulses (*p* < 0.05; Fig. 7B).

The offset difference between polarities was further assessed in a paired comparison using the “offset PE” (Fig. 7C). In accordance with the population analysis, a shift to positive offset PEs (i.e., larger offset for anodic than cathodic stimulation) was noted. Although in the bipolar configuration all offset PE values were positive, the sample size (*n* = 4) was too small for statistical testing against zero (Wilcoxon test). In the monopolar configuration, both pulse shapes resulted in significantly positive offset PEs (biphasic (*W*(62) = 500.0, *p* = 0.0125); psm (*T*(15) = 3.803, *p* = 0.0017)). The group comparison revealed significant differences in offset PEs between the stimulation modes (Kruskal–Wallis test with Dunn’s post-test: *K*(2,83) = 16.71, *p* = 0.0002, *η*^*2*^ = 0.1839) with both a significant effect of pulse shape and of electrode configuration: The offset PE was more pronounced for psm than for symmetric biphasic pulses in the monopolar configuration (post-test, *p* < 0.01; biphasic, 0.8 ± 4.4 dB; psm, 4.6 ± 5.0 dB), and the bipolar configuration yielded significantly higher offset PEs than the monopolar configuration when stimulating with symmetric biphasic pulses (post-test, *p* < 0.01; bipolar, 8.3 ± 1.0 dB). Taken together, this shows that compared to the anodic polarity in all stimulation modes, the cathodic polarity restricts the maximal available dynamic range for auditory stimulation (due to FNS).

### Relation to Cochlear Stimulation-Position and Facial Nerve Anatomy

**Fig. 8:**
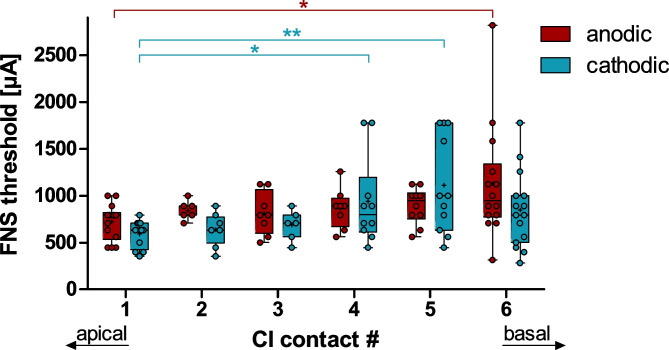
The facial nerve stimulation (FNS) threshold was significantly lower for stimulation at the most-apical contact (#1) than for basal contacts (#4–6), depending on polarity. Two-way ANOVA with post-tests: *p < .05 and **p < .01

In the symmetric biphasic-monopolar stimulation (for which we detected FNS most frequently), we also assessed potential apical-basal differences for the FNS thresholds (approximated values are not included; Fig. 8). There was an overall significant effect in the apical-basal direction (2-way ANOVA: polarity (*F*(1,112) = 1.246, *p* = 0.2667, *η*^*2*^ = 0.0110); apical-basal (*F*(5,112) = 3.822, *p* = 0.0031, *η*^*2*^ = 0.1458); interaction (*F*(5,112) = 1.519, *p* = 0.1895, *η*^*2*^ = 0.0635)). This difference was due to FNS thresholds for the most apical contact (#1) being significantly lower than for the basal electrode contacts (#4 to #6, depending on polarity; post-tests: *p* < 0.05).

**Fig. 9:**
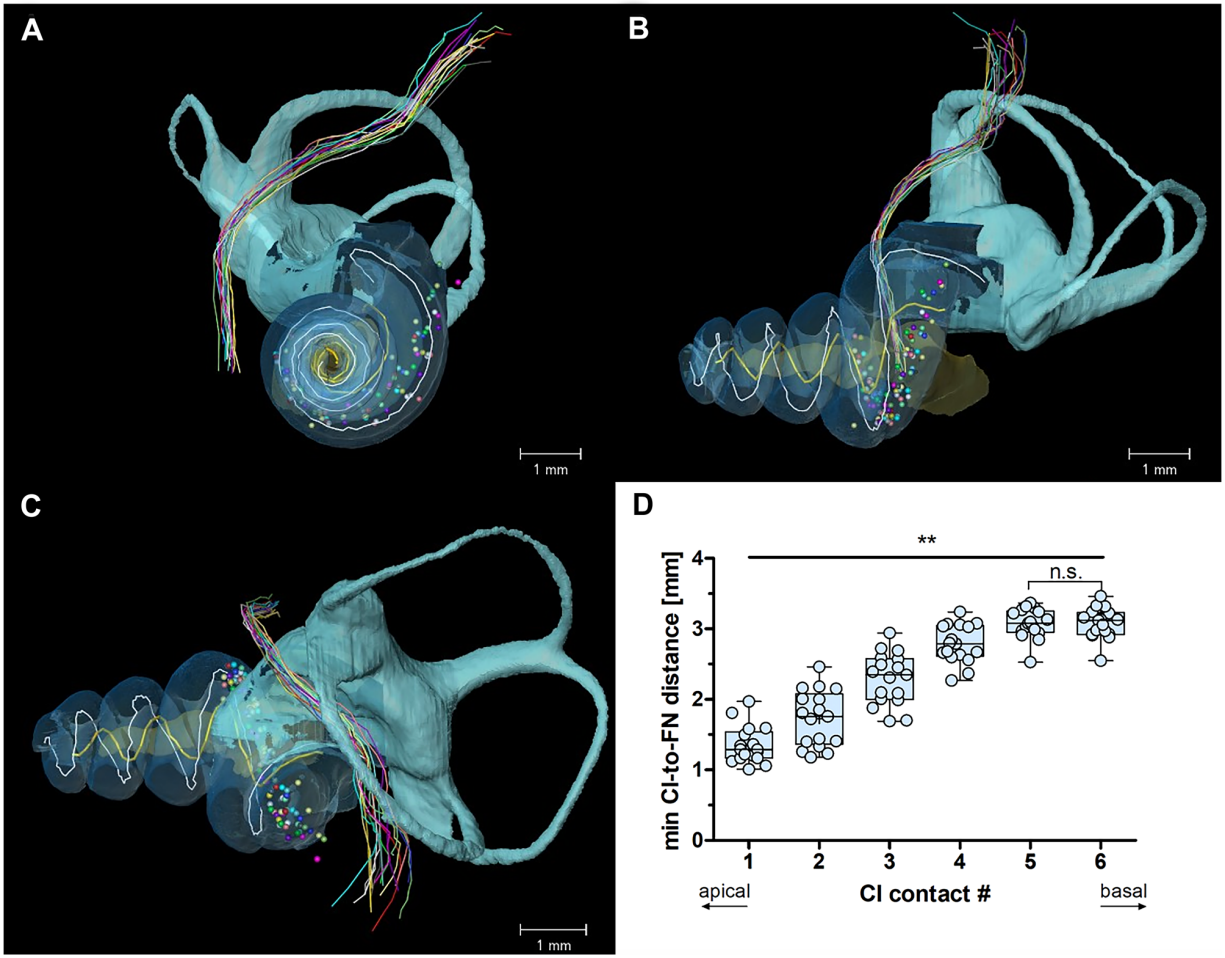
Registration of microcomputer tomography (µCT) 3-dimensional reconstructions of cochlear (CI) contacts and facial nerve (FN) tract on a µCT template. Different colors indicate corresponding FN and CI contact positions, around the template Rosenthal’s canal (yellow) and basilar membrane (white). **A–C** The template cochlear anatomy is shown as a reference: cochlea (light blue), labyrinth (cyan), and modiolus (yellow). **D** The minimal Euclidean distances of the CI electrodes to the FN (min CI-to-FN distance) increased significantly from 1.3 mm (most apical contacts) to 3.1 mm (basal contacts). Only the two most basal contacts (#5 and #6) had a similar min CI-to-FN distance (post hoc test: *p* > 0.05). One-way ANOVA with post-tests:**p < .01

We further analyzed, whether the observed apical-basal differences can be explained by differences in the distance of CI contacts to the FN tract (CI-to-FN distance). Using µCT reconstructions, we defined the minimal 3D anatomical (Euclidian) distance of the CI-electrodes to the FN (Fig. 9). In accordance with the apical-basal difference in FNS thresholds, the minimal CI-to-FN distance was smallest for contact 1 (1.35 ± 0.26 mm) and largest for the two basal-most contacts 5 and 6 (3.08 ± 0.21 mm; 1-way ANOVA: *F*(5,101) = 162.2, *p* < 0.0001, *η*^*2*^ = 0.4573; Fig. 9D). Contrary to the FNS thresholds, the CI-to-FN distance differed between all CI contacts except the two most basal ones (post-tests: *p* < 0.01). To further assess whether this discrepancy indicated that the anatomical distance is not the major defining factor for FNS thresholds, we analyzed the relationship between the two factors.

**Fig. 10:**
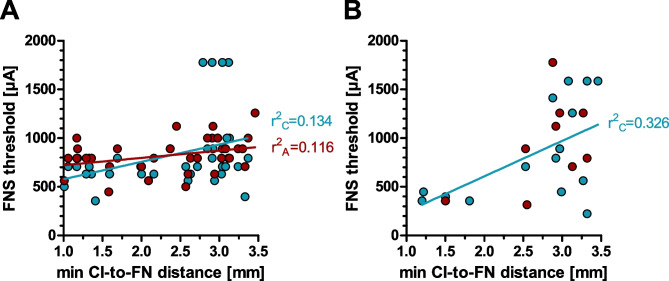
Correlations between the minimal distance of CI electrodes to FN and the FNS threshold. Linear regression lines and *r*^2^ values are given for data sets with significant correlations. The stimulation contacts were pooled for the analysis. **A** Symmetric biphasic pulses with anodic-first pulse polarity (red; *r*^2^ = 0.109, *p* = 0.044) and cathodic-first polarity (cyan; *r*^2^ = 0.189, *p* = 0.025). **B** Pseudo-monophasic (psm) pulses with anodic-second polarity (red; *p* > 0.05) and cathodic-second polarity (cyan; *r*^2^ = 0.326, *p* = 0.026)

The effect size of the relationship between anatomical distance and FNS threshold was quantified by correlating individual CI-to-FN distances with FNS thresholds for each CI contact (Fig. 10). To do this, we extended the analysis to the second stimulation in the monopolar configuration (Fig. 10B). There was a significant correlation for both symmetric biphasic (anodic (Spearman *r* = 0.4321, *p* = 0.0085), cathodic (Spearman *r* = 0.4425, *p* = 0.0078); Fig. 9A) and psm stimulation (cathodic: Pearson *r* = 0.5487, *p* = 0.0342; Fig. 9B); from about 2.5 mm distance, the thresholds increased. Only in the psm-anodic stimulation mode was the relation of the FNS threshold to the anatomy not significant (Pearson *r* = 0.3290, *p* = 0.3873). As the linear regressions yielded coefficients of determination from 0.11 to 0.33 for statistically significant correlations, no more than 11–33% of the variance in FNS threshold was explained by the variance in CI-to-FN distance.

We furthermore assessed anatomical relations separately for all three FN sections. In 90%, the overall minimal CI-to-FN distance initially used was at the “cochlear” section of the FN. To assess whether the other FN sections are a more likely origin of FNS during intra-cochlear CI stimulation, we also correlated all 3 CI-to-FN distances with the thresholds of individual CI contacts and compared the results to the correlation with the overall minimal CI-to-FN distance (Table 1). For both the anodic- and the cathodic FNS thresholds, the correlation coefficients were highest for the “cochlear” section, followed by the one for the overall minimum. This suggests that FN excitation likely occurs within the here-defined “cochlear” section of the FN. For the “cochlear” section, the linear regression (not shown) again yielded coefficients of determination ranging from 0.1 to 0.3. Thus, the anatomic distance plays a significant, but not the most important, role in FNS, explaining no more than 33% of the variance in FNS thresholds.

**Table 1:** Spearman correlation between individual CI contact thresholds (anodic and cathodic) and the minimal distance from a given CI contact to the facial nerve (CI-to-FN distance) for each contact. The CI-to-FN distance was assessed for the whole FN (overall min), as well as the three anatomical sections: cochlear, vestibular, and tympanic

	Overall min		Cochlear		Vestibular		Tympanic	
	*p*	*r*	*p*	*r*	*p*	*r*	*p*	*r*
Anodic	0.011	0.378	0.007	0.394	0.618	0.076	0.005	-0.412
Cathodic	0.004	0.400	0.002	0.429	0.055	0.2733	0.014	-0.346

## Discussion

The present study revealed two key factors underlying FNS: (1) The anodic polarity resulted in 1–4 dB higher FNS thresholds and eCAP-to-FNS offsets, and (2) the electrode configurations with focused current spread generated less FNS.

### Methodological Discussion

As our results were based on a retrospective analysis of data from experiments that were designed for different research purposes, not all stimulation modes and combinations with CI contacts were equally represented. The number of recorded eCAP input/output (I/O) functions was highest for symmetric biphasic pulses in the monopolar configuration (51%) and the lowest for symmetric biphasic pulses in the bipolar configuration (13%), with the two asymmetric biphasic (i.e., pseudo-monophasic, psm) stimulation modes falling in between (monopolar, 22%; tripolar, 14%). Nonetheless, the spread in detected FNS thresholds (Fig. 5) was very similar between symmetric biphasic pulses in both the broad-monopolar and the bipolar configuration (*SD*_anodic_: monopolar, 357 µA; bipolar, 313 µA), indicating a similarly homogenous group, irrespective of the sample size.

Furthermore, we found a relationship between the FNS thresholds and the apical-basal position of the stimulating contact along the CI (Fig. 8). Thus, for the combined analysis of all stimulation contacts (Figs. 4, 5, 6, 7 and 10), it was important that the different CI contacts were included to a similar proportion in all stimulation modes. We always included the most apical and the most basal contact of the CI in a balanced proportion with no more than 4 (0–3.8) percentage points difference between the two. Any bias would, however, not impact the polarity effect (PE; detailed discussion below), as both polarities were always presented in alternating stimulation and were, thus, equally distributed over all stimulation modes and contacts.

Occasionally, only one polarity caused FNS and the other threshold had to be “approximated”. The approximation was only used to calculate the polarity effect (i.e., threshold PE and offset PE). This was necessary, as (to assure stability of the in vivo preparation) we limited the range of current levels to prevent intense twitching of the facial muscles. Excluding such recordings would have biased our results towards those cases where the PE was small (i.e., both polarities eliciting an FNS in the given range of current levels). However, when approximating the FNS threshold as 1 dB above the stimulation range, the PE can only be underestimated and not overestimated. Based on the percentage of cases that needed approximation for PE calculation, we do not expect our results to be biased by such approximation. The percentage of approximation was similar for monopolar- and bipolar-symmetric biphasic stimulation (10%, each). Thus, approximation did not affect the reported differences due to electrode configuration. The percentage of approximation was highest when stimulating with psm pulses in the monopolar configuration (28% approximated values). Thus, the PE in psm stimulation may, overall, have been more underestimated than in the other two stimulation modes. This indicates that the true difference between pulse shapes may be even higher (not lower) than reported in this study (see below).

We found FNS in at least one recording in all of our 26 animals and in 178 of 393 recordings. This represents a high incidence (45%) compared to about 10% that was usually reported in human CI users [5, 9, 13, 24, 29]. The first potential underlying cause of this difference is related to the species: The anatomy of the inner ear and facial nerve (FN) is different in guinea pigs and humans. However, the distance and orientation of the CI contacts in the basal turn to the FN are of the same magnitude: a mean distance of 1.4 mm in humans [11] and 2.4 + 0.7 mm in guinea pigs (present study). Another difference to the abovementioned clinical reports was the use of anesthesia in our study. Thus, stimulation was also possible at high current levels, which are likely to be above the most comfortable levels (MCL) for humans. This corresponds to the intra-operative electromyographic recordings in anaesthetized human CI users, in which FNS was observed in all participants [21]. Finally, we used objective measures instead of behavioral indicators or movements of facial muscles. A study in pediatric CI users by Cushing et al. [8] reported that the incidence of subclinical FNS (i.e., myogenic responses) was much higher (59%) than the incidence of perceptual responses (39%). The incidence based on objective measures corresponds well to our study for monopolar stimulation. Thus, when assessing FNS in humans with methods similar to those used in our animal model, the reported incidences were comparable.

### Effect of the Second Phase in Symmetric Biphasic Stimulation

To reveal whether the first phase in the symmetric biphasic pulse is indeed the likely spike-initiating phase and to assess the effect of the second phase, we compared the results to those using pseudo-monophasic (psm) pulses with an initial long phase, for charge balancing, and a second, short, high-current phase for neural activation. If the long phase affected the neuronal response, this would render psm pulses more similar to symmetric biphasic pulses and lead to an underestimation of the effect of the second phase in symmetric biphasic pulses. With a 1:8 phase duration ratio, the threshold level for the long phase is ~ 6 dB higher than for the short phase, as when doubling charge by increasing the pulse duration, the threshold increases by ~ 4 dB [30–32]. We therefore expect the long phase to be ineffective within the current range applied here (i.e., ~ 7 dB above threshold).

Since for most measures there was no significant difference based on pulse shape (FNS thresholds, eCAP thresholds, eCAP-to-FNS offsets), we conclude that the prominent effect in symmetric biphasic stimulation at threshold levels is based on its leading phase. However, there was a larger PE for psm than symmetric-biphasic stimulation, with a difference of 1 dB for FNS thresholds and 4 dB for eCAP-to-FNS offsets. Thus, the second phase reduced the PE, possibly due to a quick drain for the charge released by the first phase.

### FNS Reduction by Focused and Anodic Stimulation

Both bipolar and tripolar configurations have already been shown to limit the current spread for auditory nerve stimulation [33–35]. Correspondingly, limiting the current spread by using focused configurations significantly reduced the likelihood and increased the threshold of FNS when compared to the monopolar configuration. In the bipolar configuration, the likelihood of an FNS was 20% and thus less than half of that for stimulation with a monopolar configuration (symmetric biphasic, 65%; psm, 51%). Although there was a significant higher FNS threshold for bipolar stimulation than for monopolar stimulation, the eCAP-to-FNS offset was similar to the one in monopolar configurations. In the tripolar configuration, we very rarely observed FNS (1 out of 60 cases), and thus, we did not include it in further statistical analyses. However, if we used approximated FNS threshold values (i.e., 1 dB above highest current level applied) to calculate the eCAP-to-FNS threshold (data not shown), this minimal eCAP-to-FNS offset to be expected for tripolar stimulation is already on average 1 dB higher (mean ± SD: 9 ± 5 dB) than the one for monopolar stimulation (8 ± 5 dB). Taken together, these results demonstrate that reduced current spread beyond the cochlea is a critical factor to alleviate FNS (previously suggested by [36]).

A major outcome of our study concerns the PE in the eCAP-to-FNS offset. Therefore, we first had to assess potential PE on eCAP thresholds (auditory nerve). In our previous study, including a subset of the data reported here, we reported no PE in eCAP thresholds for symmetric biphasic stimulation in control measurements [25]. This finding was confirmed here with a larger data set and extended to other stimulation modes. Previous results on the sensitivity of the guinea pig auditory nerve to the two polarities are ambiguous, showing either higher [37] or lower sensitivity to the cathodic phase [38, 39]. Correspondingly, a recent modeling study [40] suggested that biphasic pulses and/or monopolar stimulation are not suitable for detecting PE on electrically evoked neural excitation thresholds. This may be due to confounding factors, such as an apical-basal difference in susceptibility to the two polarities, which in turn may be related to differences in the orientation of the excitable structures to the current field [41]. As we found no significant PE on eCAP thresholds, we conclude that the PE in the eCAP-to-FNS offset is mainly driven by the FNS threshold PE and, thus, the characteristics of the FN and/or its anatomical relation to the CI.

In contrast to the lack of PE on eCAP thresholds, we found a significant and consistent PE on FNS thresholds. On average, we observed 1–4 dB higher FNS thresholds to anodic than to cathodic stimulation. Indications for a similar PE on FNS in mice were provided as supplement by Navntoft and colleagues (2020). The PE on FNS thresholds was further confirmed by a PE on eCAP-to-FNS offsets. The observed mean difference of up to 5 dB between anodic and cathodic stimulation might seem to be small but in fact covers the full dynamic range of single auditory nerve fibers to pulsatile CI stimulation (~ 3 dB, [42]). With our population measure (eCAP), we found dynamic ranges of ~ 7 dB (7.1 ± 3.4 dB). Thus, a 1–5-dB reduction in FNS corresponds to 14–71% of the dynamic range for electrical hearing. Furthermore, while the mean PE had the size of few dB, it covered the range of up to 12 dB (171% of the dynamic range). Taking into account the likely underestimate of the PE (see discussion on “approximation”), we expect that in a clinical setting, the beneficial effect of anodic stimulation for individual subjects will be substantial.

### The Apical-Basal Gradient in FNS

Although the anatomy and trajectory of the human FN is different from the one in guinea pigs [10], in humans, the FN is also in closest proximity to the upper basal turn of the cochlea (e.g., [43]). Based on the insertion depth, this leads to the shortest distances in the middle part of the human CI electrode array which typically occupies the upper basal turn [44, 45]. As the guinea pig CI is inserted up to the upper basal turn, the described apical-basal gradient is well in line with the distance changes described in humans.

In humans, those electrodes that are closest to the FN have, as a rule, the lowest FNS thresholds [1, 46]. Correspondingly, we found that the FNS threshold was lower for apical contacts that have the shortest distance to the FN canal. Furthermore, the minimal CI-to-FN distance correlated significantly with FNS thresholds. This corresponds to the clinical observation that the FNS incidence is significantly higher for the lateral wall than for perimodiolar/mid-scalar electrodes [13, 29]. However, our effect size was small: The variance in anatomical distance explained only 10–30% of the variance in FNS thresholds. Likewise, Gärtner et al. [18] found a reduction in FNS with appropriate change in stimulation parameters, although they were accompanied by a change from a mid-modiolar to a lateral wall electrode. Thus, the anatomical distance is one, but not the most prominent factor for reducing FNS.

The described apical-basal difference may also explain the PE in bipolar stimulation, by which a change in polarity changes the direction of current flow. Here, the stimulation term (e.g., anodic) is based on the phase of the more apical contact. As the FNS thresholds were, on average, lower for the apical contact, changing this contact to the anodic polarity is expected to reduce FNS. A similar finding was reported in humans, using the apical referencing method [45]. In this electrode configuration, a cathodic phase at the basal CI contact, with an anodic-phase at the apical reference, reduced FNS relative to the respective cathodic-first monopolar stimulation.

### “Anodic Rescue Effect” as a Potential Explanation of Clinical Findings

Based on our results in guinea pigs, we conclude that the best clinical approach to reduce FNS, within a given configuration, is through using stimulation with anodic spike-eliciting phase (e.g., anodic-leading biphasic pulses). This assumption, however, cannot be expected to directly translate to the clinics. One factor is that the eCAP threshold (used here to calculate the eCAP-to-FNS offset) are poor estimates of the MCL used clinically. The eCAP threshold underestimates the MCL, especially for high MCL thresholds [47], which may be more prone to FNS. Also the FNS threshold determined by electrophysiological methods is lower than the one reported behaviorally in CI patients [8]. With an underestimate of the threshold for both, it may be that the eCAP-to-FNS offset is a good estimate for the dynamic range from and MCL to FNS. This has, to the best of our knowledge, not yet been tested. Importantly, it is not known whether both will be similarly affected by a change in polarity. Furthermore, there are no comparable studies on FNS for guinea pigs and humans. Thus, to evaluate its translational value, we compared our results to clinical reports of FNS treatments.

The PE is most likely a relevant explanatory factor for the observed rescue effect from FNS using triphasic pulses. These findings have usually been based on a comparison of cathodic-first, biphasic to triphasic stimulation, not assessing the PE [16, 17]. When taking into account that, in the triphasic stimulation, the second, high-amplitude phase is most likely the spike-eliciting phase, these results correspond well to the “anodic rescue effect” described here, whereby only the anodic high-amplitude (i.e., cathodic-first) pulses reduced FNS. Correspondingly, the authors describe, based on single examples, that the ameliorative effect of triphasic stimulation on FNS was diminished when switching to anodic-first stimulation. Furthermore, our results provide mechanistic explanations for three very recent clinical case reports on, in total, 6 CI users with severe FNS prior to treatment [18, 22, 23]. These studies report that the change in manufacturer reduced FNS by changing the stimulation strategy from the classical cathodic-first, symmetric biphasic pulse shape in monopolar configuration to an anodic, psm pulse in a focused configuration [18, 22, 23]. Based on our results we propose that all three factors (electrode configuration, pulse shape, and pulse polarity) acted combined as “rescue factors” from FNS. Based on the good correspondence of the clinical reports with our findings in the animal model, we propose that the mechanisms for alleviating FNS, especially the “anodic rescue effect,” can be generalized from guinea pigs to humans.
